# Mobile Phone Use, Genetic Susceptibility and New-Onset Chronic Kidney Diseases

**DOI:** 10.3389/ijph.2023.1605358

**Published:** 2023-02-16

**Authors:** Yuanyuan Zhang, Yanjun Zhang, Ziliang Ye, Sisi Yang, Mengyi Liu, Qimeng Wu, Chun Zhou, Panpan He, Xianhui Qin

**Affiliations:** ^1^ Division of Nephrology, Nanfang Hospital, Southern Medical University, Guangzhou, China; ^2^ National Clinical Research Center for Kidney Disease, Guangzhou, China; ^3^ State Key Laboratory of Organ Failure Research, Guangdong Provincial Institute of Nephrology, Guangzhou, China; ^4^ Guangdong Provincial Key Laboratory of Renal Failure Research, Guangzhou, China

**Keywords:** cohort study, mobile phones uses, making or receiving calls, chronic kidney diseases, UK biobank

## Abstract

**Objective:** To examine the associations of mobile phone use and its use characteristics with new-onset CKD.

**Methods:** 408,743 participants without prior CKD in the UK Biobank were included. The primary outcome was new-onset CKD.

**Results:** During a median follow-up of 12.1 years, 10,797 (2.6%) participants occurred CKD. Compared with mobile phone non-users, a significantly higher risk of new-onset CKD was found in mobile phone users (HR = 1.07; 95% CI: 1.02–1.13). Moreover, among mobile phone users, compared with participants with weekly usage time of mobile phone making or receiving calls <30 min, a significantly higher risk of new-onset CKD was observed in those with usage time ≥30 min (HR = 1.12; 95% CI: 1.07–1.18). Moreover, participants with both high genetic risks of CKD and longer weekly usage time of mobile phones had the highest risk of CKD. Similar results were found using the propensity score matching methods. However, there were no significant associations of length of mobile phone use, and hands-free device/speakerphone use with new-onset CKD among mobile phone users.

**Conclusion:** Mobile phone use was significantly associated with a higher risk of new-onset CKD, especially in those with longer weekly usage time of mobile phones making or receiving calls. Our findings and the underlying mechanisms should be further investigated.

## Introduction

Chronic kidney disease (CKD) has a major impact on global health, both as a direct cause of morbidity and as an important risk factor for cardiovascular disease and premature death (1). In 2017, there were 697.5 million cases of CKD worldwide, with a global prevalence of 9.1% (2). CKD is largely preventable, and therefore identifying more modifiable risk factors for CKD to establish primary preventive measures has important clinical implications.

In recent years, one phenomenon worthy of our attention is the sharp increase in the number of mobile phone users around the world, with an estimated 8.2 billion subscriptions worldwide in 2020 (3). This raises the question of whether it is completely safe to make and receive calls on mobile phones, and whether there may be possible adverse health effects, especially among heavy mobile phone users. In fact, a high frequency of mobile phone use had been reported to be associated with depression symptoms, stress and sleep disturbances (4–6), all of which were related to a higher risk of CKD (7, 8). Moreover, a number of studies in animal or human cells had suggested that chronic exposure to radiofrequency electromagnetic field (RF-EMF) radiation emitted by mobile phones may increase oxidative stress, inflammatory responses and DNA damage (9, 10), and thus contribute to the pathogenesis of CKD (11–14). Accordingly, some studies in mice had found increased blood creatinine concentrations after EMF exposure of mobile phones (15, 16). As such, we speculated that mobile phone users may have a higher risk of incident CKD. However, to date, few studies have systematically assessed the relation of mobile phone use, especially some important use behaviors, such as the frequency of mobile phone use and the length starting using mobile phones, with the risk of CKD. Therefore, the relationship of mobile phone use with new-onset CKD is still uncertain.

To address these gaps in knowledge, we aimed to examine the associations of mobile phone use, and characteristics of mobile phone use, including the frequency of making or receiving calls, the length of mobile phone use and hands-free device/speakerphone use, with risk of new-onset CKD in general population, using data from the large-scale, observational UK Biobank. Moreover, since it has been established that genetic factors may contribute to the development of CKD, we further investigated the joint effect of mobile phone use and genetic susceptibility of CKD on the risk of new-onset CKD.

## Methods

### Data Source and Study Population

The UK Biobank is a large prospective, observational, population-based cohort designed to provide a resource for investigation of the genetic, environmental, and lifestyle factors associated with health and a wide range of diseases. Details of the study design and data collection have been described previously (17, 18). Briefly, the study recruited >500,000 adult participants aged 37–73 years, from 22 assessment centers across England, Wales, and Scotland from 2006 to 2010. Participants completed a touch screen questionnaire, a face-to-face interview and a series of physical measurements, and provided biological samples for laboratory analysis.

In this study, we included participants with complete information on mobile phone use characteristics, and without prior CKD [self-reported CKD diagnosis, CKD diagnosis time prior to date of baseline assessment, or estimated GFR (eGFR) < 60 mL/min/1.73 m^2^, or urine albumin: creatinine ratio (UACR) ≥30 mg/g]. Therefore, a total of 408,743 participants were enrolled in the final analysis (Supplementary Figure S1). The UK Biobank was approved by the North West Research Ethics Committee (06/MRE08/65) and all participants signed an informed consent.

### Ascertainment of Mobile Phone Use Characteristics

In the UK Biobank, mobile phone use characteristics (length of mobile phone use, weekly usage of mobile phone making or receiving calls, and hands-free device/speakerphone use to make or receive calls) were self-reported and assessed through the touchscreen questionnaire on the initial assessment visit (2006–2010).

Length of mobile phone use was assessed using the following question, “For approximately how many years have you been using a mobile phone at least once per week to make or receive calls?”, and 7 options were given to respond: “Never used mobile phone at least once per week,” “One year or less,” “Two to four years,” “Five to eight years,” “More than eight years,” “Do not know,” and “Prefer not to answer.” According to the answers to the above question, those, who reported that they have used a mobile phone at least once per week in the past, were defined as mobile phone users. Mobile phone users were further asked for weekly usage of mobile phone making or receiving calls, and hands-free device/speakerphone use with mobile phone, while others did not.

Weekly usage of mobile phones making or receiving calls was obtained using the following question, “Over the last 3 months, on average how much time per week did you spend making or receiving calls on a mobile phone?”, and 8 options were given to respond: “Less than 5 min,” “5–29 min,” “30–59 min,” “1–3 h,” “4–6 h,” “More than 6 h,” “Do not know,” and “Prefer not to answer.”

Hands-free device/speakerphone use to make or receive calls was assessed using the following question, “Over the last 3 months, how often have you used a hands-free device/speakerphone when making or receiving calls on your mobile?”, and 7 options were given to respond: “Never or almost never,” “Less than half the time,” “About half the time,” “More than half the time,” “Always or almost always,” “Do not know,” and “Prefer not to answer.”

### Definition of Genetic Risk Scores of Kidney Function

Detailed information about genotyping and quality control in the UK Biobank study has been described previously (19). Genetic risk scores (GRSs) of kidney function were calculated by 263 single nucleotide polymorphisms (SNPs) which showed independently significant genome-wide association with eGFR (20), using a weighted method (21). A higher GRS indicated a lower genetic predisposition to kidney diseases. Participants were divided into high, medium, or low genetic risks for CKD according to the tertiles of the GRS for kidney function.

### Ascertainment of Covariates

Detailed information on covariates was available through standardized questionnaires, including age, sex, ethnicities, education, smoking, alcohol drinking, income, and the uses of antihypertensive, cholesterol-lowering and glucose-lowering medications. Area-based socioeconomic status was derived from postal code of residence by using the Townsend deprivation score. BMI was calculated from weight (kg)/height(m) (2). Prevalent diabetes at baseline was identified through multiple procedures considering type of diabetes and sources of the diagnosis (22).

The UK biobank blood collection sampling procedures have previously been described and validated (23). Biochemical assays were performed at a dedicated central laboratory. The estimated glomerular filtration rate (eGFR) was calculated by Chronic Kidney Disease Epidemiology Collaboration equation (24).

### Study Outcome

The study outcome was new-onset CKD, which was defined according to the International Classification of Diseases (ICD) edition 9 code of 585 and 5859, ICD edition 10 code of N12.0, N13.1, N13.2, N18.0, N18.3, N18.4, N18.5, N18.8, and N18.9, and the Office of Population Censuses and Surveys Classification of Interventions and Procedures, version 4 (OPCS-4) code of M01 (Supplementary Table S1). The follow-up for each participant was calculated from the date of first assessment until the first date of new-onset CKD, date of death, date of lose to follow-up, or the end of follow-up, whichever came first.

### Statistical Analysis

Baseline characteristics of study participants were presented as mean ± standard deviation (SD) for continuous variables and proportions for categorical variables. Comparisons of the characteristics according to the weekly usage time of mobile phones making or receiving calls (<5 min, 5–29 min, 30–59 min, 1–3 h, 4–6 h, and >6 h) were performed by chi-square tests for categorical variables and one-way analysis of variance (ANOVA) for continuous variables among mobile phone users.

Cox proportional hazards models were used to investigate the relations of mobile phone use [users (mobile phone use at least once per week to make or receive calls) vs. non-users] with new-onset CKD in the total participants, and the associations of weekly usage time of mobile phones making or receiving calls, length of mobile phone use (≤1 year, 2–4 years, 5–8 years, and >8 years) and hands-free device/speakerphone use to make or receive calls (Never or almost never, Less than half the time, About half the time, More than half the time, and Always or almost always) with new-onset CKD in mobile phone users. Model 1 adjusted for age and sex. Model 2 adjusted for age, sex, BMI, ethnicities, Townsend deprivation index, income, education, smoking, alcohol drinking, systolic blood pressure (SBP), triglycerides, low-density lipoprotein (LDL) cholesterol, high-density lipoprotein (HDL) cholesterol, C-reactive protein, glycosylated hemoglobin (HbA1c), eGFR, uses of antihypertensive medications, cholesterol-lowering medications and glucose-lowering medications. Model 3 included all the covariates in Model 2 plus mutually adjustments for different characteristics of mobile phone use (weekly usage time of mobile phones making or receiving calls, length of mobile phone use and hands-free device/speakerphone use to make or receive calls) among mobile phone users. The proportional hazards assumptions for the Cox model were tested using Schoenfeld residuals method and no violation of this assumption was detected.

Moreover, we also estimated the joint effect of weekly usage time of mobile phones making or receiving calls and genetic risks of CKD on the risk of new-onset CKD using weekly usage time <5 min with a low genetic risk of CKD as reference. Stratified analysis was conducted to assess potential modification effects of weekly usage time of mobile phones making or receiving calls (<30 or ≥30 min) with new-onset CKD according to age (<60 or ≥60 years), sex, BMI (<30 or ≥30 kg/m^2^), smoking status (never, former or current), SBP (<140 or ≥140 mmHg), eGFR (<90 or ≥90 mL/min/1.73 m^2^), diabetes (no or yes), length of mobile phone use, and hands-free device/speakerphone use to make or receive calls. Interactions were examined by likelihood ratio testing.

We also conducted a series of sensitively analysis to assess the robustness of the results. First, physical activity, healthy sleep scores (25), healthy diet scores (26), self-reported depression, total mental health complaints (27) and genetic risk scores of kidney function were further adjusted for. Second, we excluded those who occurred new-onset CKD during the first 2 years of follow-up. Third, propensity score matching methods were used to further evaluated our results. A non-parsimonious propensity score using variables that might affect mobile phones uses/weekly usage time of mobile phone or new-onset chronic kidney disease was developed to predict the likelihood a participant would be in the different status of mobile phone uses (no or yes), or in the different length of weekly usage time of mobile phone making or receiving calls (<30 or ≥30 min). Participants were matched 1:1 based on propensity scores. An automated balance optimization method using the function Match (in package Matching) in R and a caliper of 0.2 were used for matching. Standardized differences of post-matched participant characteristics ≤10% between the 2 groups was considered to be balanced.

A two-tailed *p* < 0.05 was considered to be statistically significant in all analyses. Analyses were performed using R software (version 4.1.3, http://www.R-project.org/).

## Results

### Baseline Characteristics of the Participants

As illustrated in the flow chart (Supplementary Figure S1), a total of 408,743 participants were included in the current study. Of those, 348,602 participants were mobile phone users, and 60,141 were mobile phone non-users. The mean (SD) age was 56.3 (8.1) years, and 188,756 (46.2%) were male.

Compared with mobile phone non-users, mobile phone users were younger, more likely to be smokers, had higher BMI, eGFR, income levels, lower SBP levels, and lower usage of antihypertensive medications, cholesterol-lowering medications and glucose-lowering medications (Supplementary Table S2).

Moreover, among mobile phone users, participants with longer weekly usage time of mobile phones making or receiving calls were younger, more likely to be male, current smokers and to use hands-free device/speakerphone, had lower SBP levels, lower use of antihypertensive and cholesterol-lowering medications, higher Townsend deprivation index, income, BMI, eGFR, TG levels, and higher length of mobile phone use (Table 1).

**TABLE 1:** Baseline characteristics of mobile phone users according to weekly usage time of mobile phones making or receiving calls (UK, 2006–2010).

Baseline characteristics^a^	Weekly usage time of mobile phone making or receiving calls						*p*-value
	<5mins	5–29 min	30–59 min	1–3 h	4–6 h	>6 h	
N	71,891	135,929	60,562	50,273	14,797	15,150	
Age, years	58.4 ± 7.6	56.5 ± 7.9	54.8 ± 7.8	53.0 ± 7.7	51.6 ± 7.4	50.4 ± 7.0	<0.001
Male, n (%)	31,896 (44.4)	57,623 (42.4)	28,178 (46.5)	25,839 (51.4)	8,059 (54.5)	8,678 (57.3)	<0.001
White, n (%)	69,367 (96.5)	130,459 (96.0)	57,356 (94.7)	46,611 (92.7)	13,554 (91.6)	13,662 (90.2)	<0.001
Body mass index, kg/m^2^	27.0 ± 4.5	27.2 ± 4.6	27.5 ± 4.7	27.7 ± 4.7	28.1 ± 4.8	28.4 ± 4.9	<0.001
Systolic blood pressure, mmHg	139.1 ± 18.5	137.3 ± 18.2	135.7 ± 17.9	134.6 ± 17.4	134.0 ± 17.0	133.3 ± 16.8	<0.001
Diastolic blood pressure, mmHg	82.1 ± 9.9	82.0 ± 9.9	81.9 ± 10.0	82.1 ± 10.1	82.3 ± 10.2	82.5 ± 10.2	<0.001
Townsend deprivation index	−1.7 ± 2.9	−1.5 ± 3.0	−1.2 ± 3.1	−1.1 ± 3.2	−1.0 ± 3.2	−1.0 ± 3.3	<0.001
College or University degree, n (%)	23,011 (32.3)	45,766 (33.9)	20,593 (34.2)	16,887 (33.8)	4,707 (32.1)	4,528 (30.1)	<0.001
Current smoker, n (%)							<0.001
Never	40,846 (57.0)	74,406 (54.9)	31,569 (52.3)	26,021 (51.9)	7,731 (52.4)	7,726 (51.2)	
Former	25,016 (34.9)	48,569 (35.8)	21,994 (36.4)	17,468 (34.8)	4,828 (32.7)	4,751 (31.5)	
Current	5,796 (8.1)	12,562 (9.3)	6,817 (11.3)	6,640 (13.2)	2,188 (14.8)	2,622 (17.4)	
Alcohol drinking, n (%)							<0.001
Never	5,457 (7.6)	8,794 (6.5)	3,917 (6.5)	3,361 (6.7)	1,042 (7.0)	1,194 (7.9)	
<1 time per week	16,291 (22.7)	29,494 (21.7)	12,535 (20.7)	10,471 (20.8)	3,138 (21.2)	3,244 (21.4)	
1–4 time per week	35,139 (48.9)	69,528 (51.2)	31,253 (51.6)	25,801 (51.4)	7,687 (52.0)	7,576 (50.1)	
Daily or almost daily	14,959 (20.8)	28,043 (20.7)	12,828 (21.2)	10,612 (21.1)	2,923 (19.8)	3,122 (20.6)	
Income, n (%)							<0.001
Not to answer/Do not know	10,699 (14.9)	17,338 (12.8)	7,096 (11.7)	5,254 (10.5)	1,513 (10.2)	1,477 (9.8)	
Less than 18,000	15,608 (21.8)	24,275 (17.9)	9,591 (15.9)	7,110 (14.2)	1934 (13.1)	1892 (12.5)	
18,000 to 30,999	17,455 (24.3)	30,594 (22.6)	12,212 (20.2)	9,121 (18.2)	2,409 (16.3)	2,269 (15.0)	
31,000 to 51,999	15,581 (22.1)	32,255 (23.8)	14,763 (24.4)	12,442 (24.8)	3,581 (24.2)	3,637 (24.0)	
52,000 to 100,000	10,112 (14.1)	25,002 (18.4)	13,001 (21.5)	12,197 (24.3)	3,889 (26.3)	4,171 (27.6)	
Greater than 100,000	1971 (2.7)	6,193 (4.6)	3,767 (6.2)	4,053 (8.1)	1,448 (9.8)	1,682 (11.1)	
LDL cholesterol, mmol/L	3.6 ± 0.9	3.6 ± 0.9	3.6 ± 0.9	3.6 ± 0.8	3.6 ± 0.8	3.6 ± 0.8	0.003
HDL cholesterol, mmol/L	1.5 ± 0.4	1.5 ± 0.4	1.4 ± 0.4	1.4 ± 0.4	1.4 ± 0.4	1.4 ± 0.4	<0.001
Triglycerides, mmol/L	1.7 ± 1.0	1.7 ± 1.0	1.7 ± 1.0	1.8 ± 1.1	1.8 ± 1.1	1.9 ± 1.2	<0.001
HbA1c, %	5.4 ± 0.6	5.4 ± 0.5	5.4 ± 0.6	5.4 ± 0.6	5.4 ± 0.6	5.4 ± 0.6	<0.001
C-reactive protein, mg/L	2.5 ± 4.1	2.5 ± 4.1	2.5 ± 4.2	2.5 ± 4.0	2.5 ± 4.0	2.6 ± 4.0	0.006
eGFR, mL/min/1.73m2	90.3 ± 11.7	91.6 ± 11.9	92.8 ± 11.9	94.1 ± 12.0	94.8 ± 12.1	95.4 ± 12.2	<0.001
Antihypertensive medications use, n (%)	15,392 (21.5)	26,086 (19.3)	10,364 (17.2)	7,770 (15.6)	2056 (14.0)	2004 (13.4)	<0.001
Cholesterol lowering medications use, n (%)	13,046 (18.3)	21,687 (16.0)	8,855 (14.7)	6,502 (13.0)	1742 (11.9)	1717 (11.4)	<0.001
Glucose-lowering medications use, n (%)	2,199 (3.1)	3,791 (2.8)	1820 (3.0)	1,421 (2.8)	409 (2.8)	439 (2.9)	0.005
Length of mobile phone use, n (%)							<0.001
One year or less	5,639 (7.8)	3,930 (2.9)	742 (1.2)	360 (0.7)	64 (0.4)	49 (0.3)	
Two to four years	22,152 (30.8)	31,367 (23.1)	9,805 (16.2)	5,512 (11.0)	1,229 (8.3)	804 (5.3)	
Five to eight years	26,707 (37.1)	53,784 (39.6)	22,447 (37.1)	16,342 (32.5)	4,112 (27.8)	3,129 (20.7)	
More than eight years	17,393 (24.2)	46,848 (34.5)	27,568 (45.5)	28,059 (55.8)	9,392 (63.5)	11,168 (73.7)	
Hands-free device/speakerphone use, n (%)							<0.001
Never or almost never	68,370 (95.1)	120,505 (88.7)	47,696 (78.8)	33,662 (67.0)	8,127 (54.9)	6,973 (46.0)	
Less than half the time	1903 (2.6)	9,095 (6.7)	7,274 (12.0)	8,843 (17.6)	3,133 (21.2)	3,285 (21.7)	
About half the time	673 (0.9)	2,960 (2.2)	2,694 (4.4)	3,506 (7.0)	1,515 (10.2)	1,673 (11)	
More than half the time	278 (0.4)	1,378 (1.0)	1,325 (2.2)	2031 (4.0)	951 (6.4)	1,338 (8.8)	
Always or almost always	667 (0.9)	1991 (1.5)	1,573 (2.6)	2,231 (4.4)	1,071 (7.2)	1881 (12.4)	

^a^ The results are presented as Mean ± SD or n (%).

Abbreviations: eGFR, estimated glomerular filtration rate; HbA1c, glycosylated hemoglobin, HDL cholesterol, high density lipoprotein cholesterol; LDL cholesterol, low density lipoprotein cholesterol.

### Relation of Mobile Phone Use and New-Onset CKD in Total Participants

During a median follow-up duration of 12.1 years, a total of 10,797 (2.6%) participants occurred new-onset CKD. Compared with mobile phone non-users, a significantly higher risk of new-onset CKD was found in mobile phone users (HR, 1.07; 95% CI: 1.02–1.13) (Table 2).

**TABLE 2:** Association between mobile phones uses (vs. non-users) and new-onset chronic kidney diseases among total participants, and relations of different mobile phone use characteristics with new-onset chronic kidney diseases in mobile phones users (UK, 2006–2010).

Mobile phone use characteristics	N	Events (%)	Model 1^a^		Model 2^b^		Model 3^c^	
			HR (95% CI)	*p*-value	HR (95% CI)	*p*-value	HR (95% CI)	*p*-value
Total participants (N = 408,743)								
Mobile phone users								
No	60,141	2067 (3.4)	Ref		Ref		-	
Yes	348,602	8,730 (2.5)	1.07 (1.02, 1.12)	0.006	1.07 (1.02, 1.13)	0.009	-	-
Mobile phone users (N = 348,602)								
Length of mobile phone use								
≤1 year	10,784	389 (3.6)	Ref		Ref		Ref	
2–4 years	70,869	2005 (2.8)	0.88 (0.79, 0.98)	0.016	0.94 (0.83, 1.06)	0.331	0.93 (0.82, 1.05)	0.250
5–8 years	126,521	3,083 (2.4)	0.83 (0.75, 0.93)	<0.001	0.94 (0.83, 1.06)	0.301	0.92 (0.81, 1.03)	0.162
>8 years	140,428	3,253 (2.3)	0.88 (0.79, 0.97)	0.014	1.01 (0.90, 1.14)	0.848	0.97 (0.86, 1.09)	0.590
*P* for trend			0.252		0.067		0.387	
Weekly usage time of mobile phone making or receiving calls								
<5 min	71,891	2,138 (3.0)	Ref		Ref		Ref	
5–29 min	135,929	3,455 (2.5)	1.02 (0.96, 1.08)	0.508	0.99 (0.94, 1.05)	0.808	0.99 (0.94, 1.05)	0.814
30–59 min	60,562	1,464 (2.4)	1.15 (1.08, 1.23)	<0.001	1.09 (1.02, 1.18)	0.018	1.09 (1.01, 1.17)	0.026
1–3 h	50,273	1,076 (2.1)	1.21 (1.13, 1.31)	<0.001	1.14 (1.05, 1.24)	0.002	1.13 (1.04, 1.23)	0.005
4–6 h	14,797	281 (1.9)	1.24 (1.09, 1.41)	<0.001	1.17 (1.02, 1.34)	0.025	1.15 (1.00, 1.32)	0.047
>6 h	15,150	316 (2.1)	1.58 (1.40, 1.79)	<0.001	1.31 (1.14, 1.49)	<0.001	1.28 (1.11, 1.47)	<0.001
*P* for trend			<0.001		<0.001		<0.001	
Categories								
<30 min	207,820	5,593 (2.7)	Ref		Ref		Ref	
≥30 min	140,782	3,137 (2.2)	1.20 (1.14, 1.25)	<0.001	1.14 (1.08, 1.19)	<0.001	1.12 (1.07, 1.18)	<0.001
Hands-free device/speakerphone use								
Never or almost never	285,333	7,427 (2.6)	Ref		Ref		Ref	
Less than half the time	33,533	680 (2.0)	1.04 (0.96, 1.13)	0.289	1.08 (0.99, 1.18)	0.089	1.03 (0.94, 1.13)	0.485
About half the time	13,021	259 (2.0)	1.07 (0.94, 1.21)	0.297	1.10 (0.96, 1.26)	0.176	1.04 (0.90, 1.19)	0.609
More than half the time	7,301	138 (1.9)	1.04 (0.88, 1.24)	0.613	1.06 (0.88, 1.28)	0.518	0.99 (0.82, 1.19)	0.903
Always or almost always	9,414	226 (2.4)	1.18 (1.03, 1.34)	0.017	1.13 (0.98, 1.30)	0.096	1.06 (0.92, 1.23)	0.429

^a^ Model 1: adjusted for age, sex.

^b^ Model 2: adjusted for age, sex, body mass index, ethnicities, Townsend deprivation index, income, education, smoking status, alcohol drinking, systolic blood pressure, LDL cholesterol, HDL cholesterol, triglycerides, HbA1c, eGFR, C-reactive protein, antihypertensive medications use, cholesterol lowering medications use, glucose-lowering medications use.

^c^ Model 3: adjusted for covariates in Model 2 plus mutually adjustments for different behavior of using mobile phone.

### Relation of Weekly Usage Time of Mobile Phones Making or Receiving Calls With New-Onset CKD Among Mobile Phones Users

Overall, there were no significant associations of the length of mobile phone use, and hands-free device/speakerphone use with new-onset CKD among mobile phone users (Table 2).

However, among mobile phone users, compared with participants with weekly usage time of mobile phones making or receiving calls <5 min, significantly higher risks of new-onset CKD were observed in those with usage time 30–59 min (HR, 1.09; 95% CI: 1.01–1.17), 1–3 h (HR, 1.13; 95% CI: 1.04–1.23), 4–6 h (HR, 1.15; 95% CI: 1.00–1.32) and >6 h (HR, 1.28; 95% CI: 1.11–1.47) (*P* for trend <0.001). Consistently, compared with participates with weekly usage time of mobile phone making or receiving calls <30 min, a significantly higher risk of new-onset CKD was found in those with weekly usage time ≥30 min (HR, 1.12; 95% CI: 1.07–1.18) (Table 2).

Further adjustments for physical activity, healthy diet scores, healthy sleep scores, self-reported depression, total mental health complaints and genetic risk scores of kidney function (Supplementary Table S3), or excluding those who occurred new-onset CKD during the first 2 years of follow-up (Supplementary Table S4) did not substantially change the association of weekly usage time of mobile phones making or receiving calls with new-onset CKD.

Moreover, a significantly higher risk of new-onset CKD was found in participants with diabetes (vs. without diabetes, adjusted HR, 2.06; 95% CI: 1.91–2.23), hypertension (vs. without hypertension, adjusted HR, 1.47; 95% CI: 1.37–1.57), and high genetic risks of CKD (vs. low genetic risks, adjusted HR,1.09; 95% CI: 1.03–1.16) (Supplementary Table S5).

### Propensity Scores Analysis

After propensity score matching, 101,816 participants (50,908 in each group) were included in the analysis for mobile phone uses (non-users vs. users) and new-onset CKD, and 183,068 participants (91,534 in each group) were included in the analysis for weekly usage time of mobile phone making or receiving calls (<30 min vs. ≥30 min) and new-onset chronic kidney disease. All the post-matched participant characteristics were highly balanced (Supplementary Figures S2, S3). Consistently, a significantly higher risk of new-onset CKD was found in mobile phone users (vs. non-users, HR, 1.11; 95% CI: 1.04–1.19), and in those with weekly usage time of mobile phones making or receiving calls ≥30 min (vs. <30 min, HR, 1.10; 95% CI: 1.03–1.17) (Supplementary Table S6).

### Joint Effect of Weekly Usage Time of Mobile Phones Making or Receiving Calls and Genetic Risks of CKD on New-Onset CKD Among Mobile Phones Users

**FIGURE 1:**
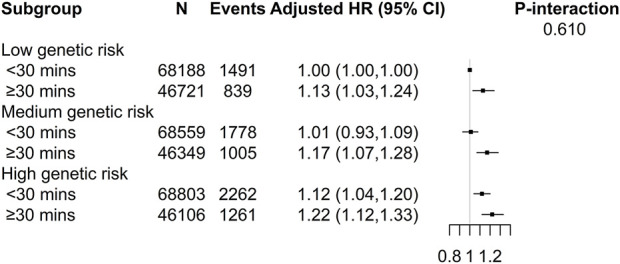
Joint effect of weekly usage time of mobile phones making or receiving calls and genetic risks of kidney diseases with new-onset chronic kidney disease among mobile phones users (UK, 2006–2010). *Adjusted for age, sex, body mass index, ethnicities, Townsend deprivation index, income, education, smoking status, alcohol status, systolic blood pressure, diastolic blood pressure, total cholesterol, triglycerides, HDL cholesterol, HbA1c, eGFR, antihypertensive medications use, cholesterol lowering medications use, hypoglycemic medications use, length of mobile phone use, and hands-free device/speakerphone use.

Compared with those with a low genetic risk of CKD and weekly usage time of mobile phones making or receiving calls <30 min, participants with a high genetic risk of CKD and weekly usage time ≥30 min had the highest risk of CKD (HR, 1.22, 95% CI, 1.12–1.33; Figure 1), though the interaction between weekly usage time of mobile phones making or receiving calls and genetic risks of CKD was not significant (*P*-interaction = 0.610).

### Stratified Analyses

**FIGURE 2:**
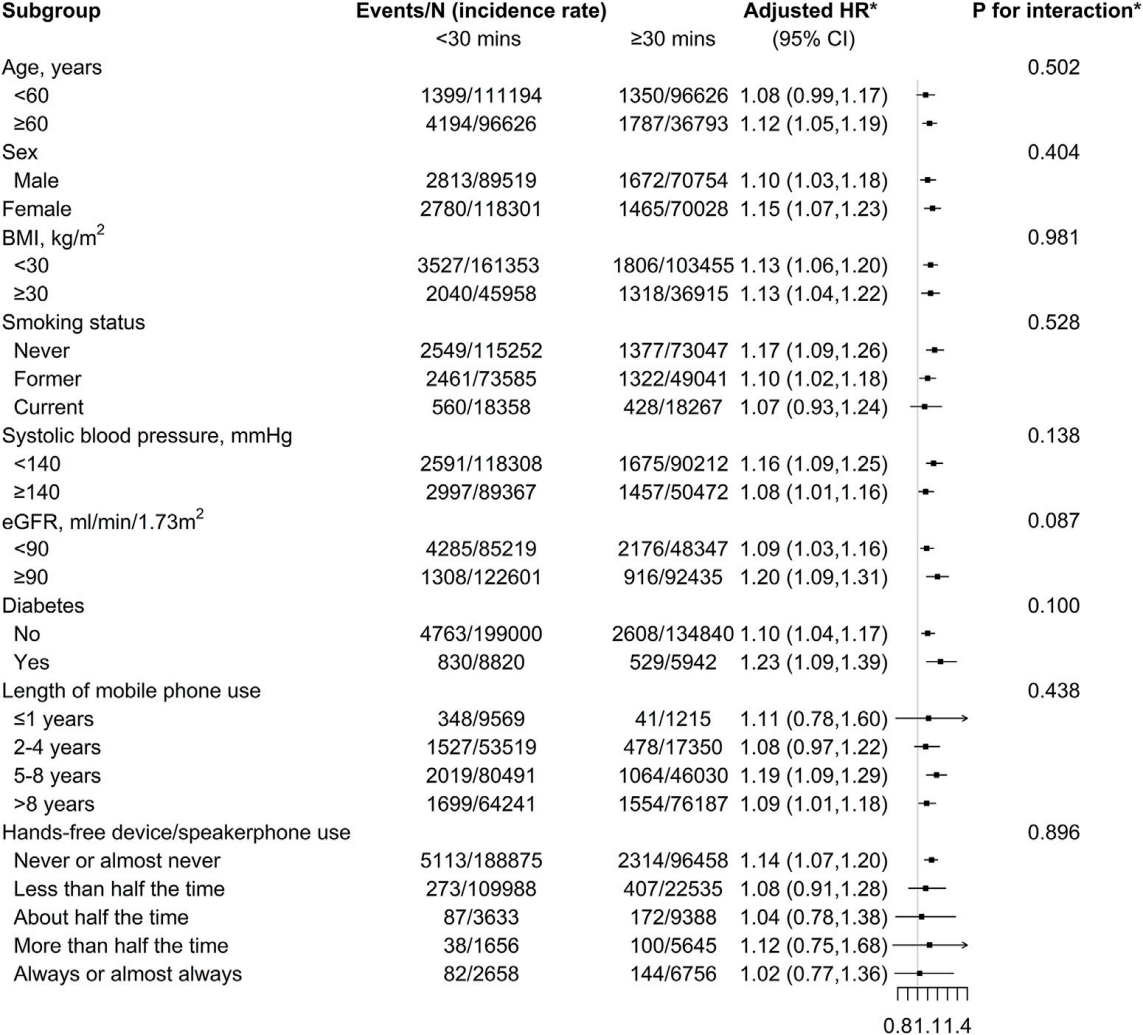
Stratified analyses of the association between weekly usage time of mobile phone making or receiving calls and new-onset chronic kidney disease among mobile phone users (UK, 2006–2010). ^*^Adjusted, if not stratified, for age, sex, body mass index, ethnicities, Townsend deprivation index, income, education, smoking status, alcohol status, systolic blood pressure, diastolic blood pressure, total cholesterol, triglycerides, HDL cholesterol, Hba1C, eGFR, antihypertensive medications use, cholesterol lowering medications use, hypoglycemic medications use, length of mobile phone use, and hands-free device/speakerphone uses.

Stratified analyses were conducted to further evaluate the association between weekly usage time of mobile phones making or receiving calls (<30 vs. ≥30 min) and new-onset CKD in various subgroups (Figure 2).

None of the variables, including age, sex, BMI, smoking, SBP, eGFR, diabetes, length of mobile phone use, and hands-free device/speakerphone uses, significantly modify the association between weekly usage time of mobile phones and new-onset CKD (all *p* values for interaction >0.05).

## Discussion

In this large, prospective cohort study, we first reported that mobile phones use was significantly related to a higher risk of new-onset CKD. Moreover, among mobile phone users, there was a significantly positive association of weekly usage time of mobile phones making or receiving calls with new-onset CKD, regardless of the genetic risks of CKD. However, there were no significant associations of the length of mobile phone use, and hands-free device/speakerphone use with new-onset CKD among mobile phone users.

In recent years, mobile phones have become a fundamental part of our social lives. Of note, although a previous study in mice found that exposed to 40 or 60 min of mobile phones radiation daily would significantly increase total leukocyte count and serum creatinine values, and exposed to 60 min of mobile phone radiation daily would cause interstitial inflammation of the kidney (15), to date, few studies had examined the prospective association of mobile phone use with risk of CKD. Our current study addressed the knowledge gap between mobile phone use and new-onset CKD in a timely manner, by considering both mobile phone use and a range of important mobile phone use characteristics.

We first found that there was a positive relationship between weekly usage time of mobile phones making or receiving calls and new-onset CKD. Our findings are biologically plausible. First, forearm lifting and static holding of the phone, a typical position for making and receiving calls, may increase sympathetic activity (28, 29) and lead to short-term increases in plasma adrenomedullin levels (30), thereby promoting myocardial contractility, resulting in systemic vasoconstriction and increased blood pressure, which was related to an increased risk of CKD. Second, a high frequency of mobile phone use was associated with depression symptoms, stress and sleep disturbances (4–6), all of which have been reported to be related to a higher risk of CKD (7, 8). However, further adjustments for healthy sleep scores, self-reported depression, and total mental health complaints did not substantially change our findings, suggesting that these factors also did not fully explain the association between weekly usage time of mobile phones making or receiving calls and new-onset CKD. Third, some previous studies have shown that the RF-EMF radiation of mobile phone use can cause a number of harmful effects at the molecular and cellular levels, including oxidative stress, inflammation and DNA damage (9, 10), and thus contribute to the development of CKD (6–8). Consistently, several studies in mice had reported that EMF exposure of mobile phones significantly increased serum creatinine concentrations (15, 16) and led to the kidney interstitial inflammation that caused marked mononuclear cellular infiltration (15). Of note, findings derived from animal experiments with rats receiving full-body high exposure to EMF may not be directly extrapolated to humans. However, Chen et al. (31) and Zhang et al. (32) reported that increasing daily calling time was significantly associated with decreased sperm concentration and total count, potentially due to increased oxidative stress and DNA fragmentation and apoptosis caused by RF-EMF radiation. Cho et al. (33) found that mobile phone call duration was significantly associated with the severity of headaches. A recent meta-analysis in human studies (34) also showed that increasing mobile phone use was associated with a higher risk of DNA damage. Due to the observed detrimental effects of calling time and RF-EMF radiation on a range of health outcomes in humans among the above studies (31–34), we speculate that RF-EMF radiation of mobile phones may also play a role in the development of CKD. Nevertheless, a previous cohort study in Denmark found that there were no significant associations between mobile phone use and tumor risks (35–38), except for a significant increase in the risk of smoking-related cancers in women and a significant decrease in the risks of smoking-related cancers in men (38). However, this study may have exposure misclassification due to the use of subscription information rather than mobile phone use. The UK Million Women Study also showed that mobile phone use was not associated with the risk of all intracranial central nervous system tumors or non-CNS cancers (39, 40). However, this study only included middle-aged women, and did not consider some important confounding factors, including menopause status, family history of cancers, oral contraceptive pill use, and age at menarche, etc. Overall, to date, there is no established biological mechanism for our findings. More studies are needed to confirm our results and further clarify the underlying biological mechanisms.

In addition, our study showed that there were no significant association of the length of mobile phone use with new-onset CKD among mobile phone users. Consistently, Chen et al. (31) found daily talking time on the cell phone was negatively associated with sperm concentration and total count; while there were no significant association between daily duration of having the cell phones on and sperm quality parameters. Our results further indicated that it is the calling time of mobile phones, rather than how long having mobile phones, that determined the impact of mobile phones use on new-onset CKD. In other words, even if the participants used mobile phones for a long time, they may possibly not have an increased risk of developing CKD if they made or received calls for less than 30 min per week. In fact, many of the previous studies (31–33) that have found adverse health effects associated with mobile phone use were based on exposure to the calling duration. Notably, as an observational study, our study was just hypothesis generating and should be confirmed in more studies.

Of note, we also found that use of hands-free device/speakerphone did not affect the risk of new-onset CKD, suggesting that holding mobile phones close to the head was not a determinant of its detrimental effects on health. Even a hands-free device/speakerphone was used when making or receiving calls, the mobile phones remained close to the body, although it may not be near the kidneys, and therefore the RF-EMF radiation of mobile phones could still affect the hematological parameters, induce the production of mitochondrial reactive oxygen species (ROS) (41) and cause DNA damage, thus increasing the risk of CKD.

The major strengths of the current study include a prospective design, a large sample size, a long follow-up, and the ability to simultaneously consider multiple mobile phone use characteristics. Our study also had some limitations. First, in the current study, the information on mobile phone use were based on the questionnaires at baseline, and the weekly usage time making or receiving calls was only considered for the 3 months prior to the interview. As such, we could not evaluate the association between cumulative exposure of mobile phone use or lifetime usage of mobile phones and new-onset CKD. However, due to the continuous increase in the number of mobile phone users over the years, mobile phone non-users may possibly have used mobile phones subsequently. Moreover, with the pace of work and life accelerating worldwide, mobile phone users are likely to spend more time making or receiving calls. Therefore, our study may possibly underestimate the relationship of mobile phone use and weekly usage time making or receiving calls with the risk of CKD. Second, the participants were predominantly of European descent and were healthier than the general UK population (42), which may limit the generalizations of the results to other populations. Third, as an observational study, although a range of possible confounders had been adjusted for, we cannot completely exclude the possibility of residual confounding due to unmeasured or unknown factors. As such, it is necessary to confirm our findings further in more studies.

In summary, mobile phone use was significantly associated with a higher risk of new-onset CKD, especially in those with longer weekly usage time of mobile phones making or receiving calls, among the general population. Of note, there is no established biological mechanism for the results. Our findings and the underlying mechanisms should be further investigated in more studies. If further confirmed, our study highlights the importance of reducing usage time of mobile phones making or receiving calls for primary prevention of CKD in the general population.
