# Supplementary material for: Mobile Phone Use, Genetic Susceptibility and New-Onset Chronic Kidney Diseases
Source: Int J Public Health. 2023 Feb 16;68:1605358. doi: 10.3389/ijph.2023.1605358 (PMC9977800; doi:10.3389/ijph.2023.1605358)
Supplement: Supplementary file 1 [file DataSheet1.doc]

**Mobile phones use, genetic susceptibility and new-onset chronic kidney diseases Running head:** Mobile phones uses and new-onset CKD

502,414 participants enrolled in the UK Biobank

Excluded n=81,616

Having or missing values on chronic kidney disease at baseline

420,798 participants included

Excluded n=12,055

Missing values on questionnaires of mobile phone use characteristics

408,743 participants included for current analyses

**Supplementary Figure 1. Flow chart of the participants (UK, 2006-2010)**

**
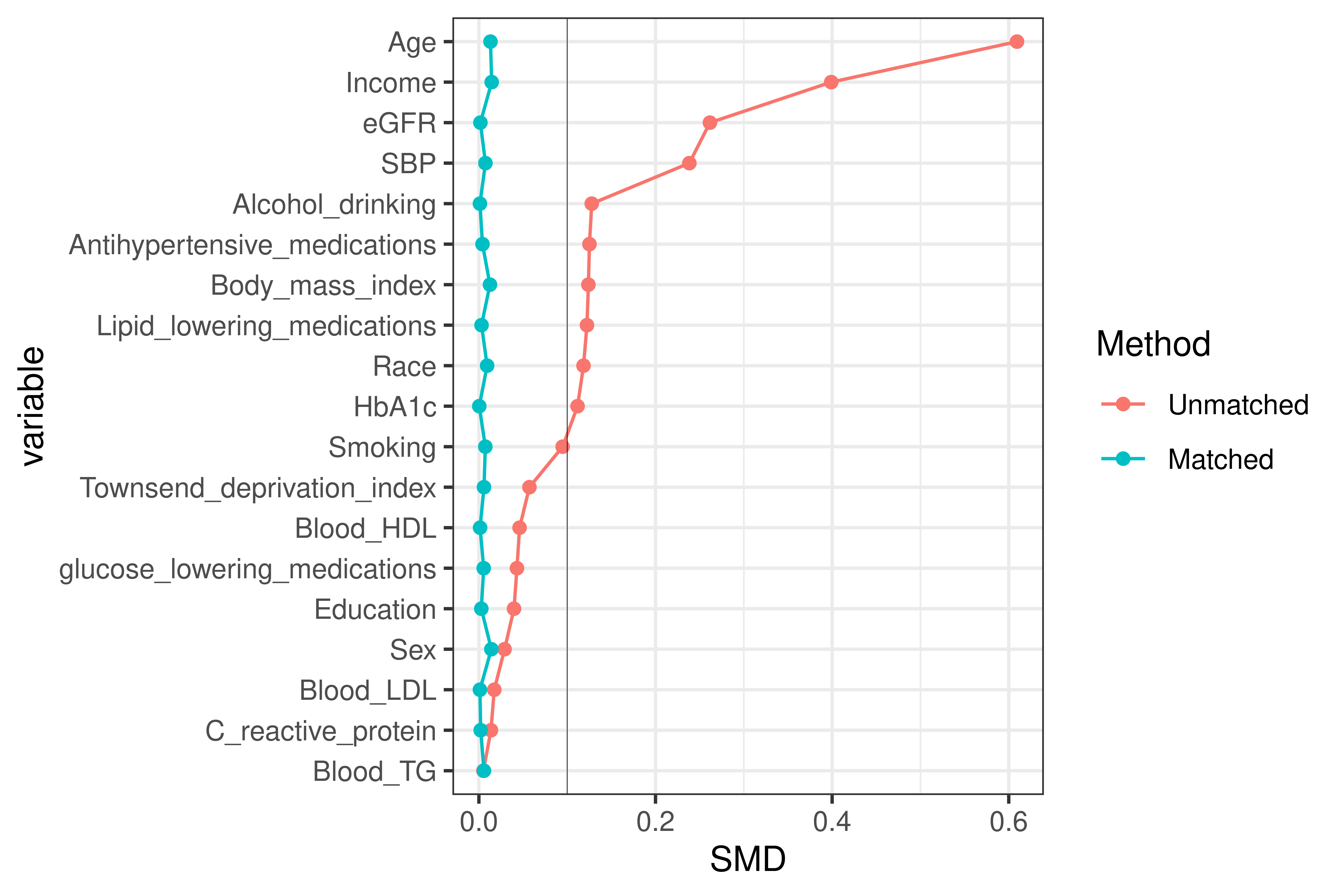
**

**Supplementary Figure 2. Standardized differences of participant characteristics before and after matched according to propensity scores by mobile phones use status (non-users *vs.* users) (UK, 2006-2010)**

**
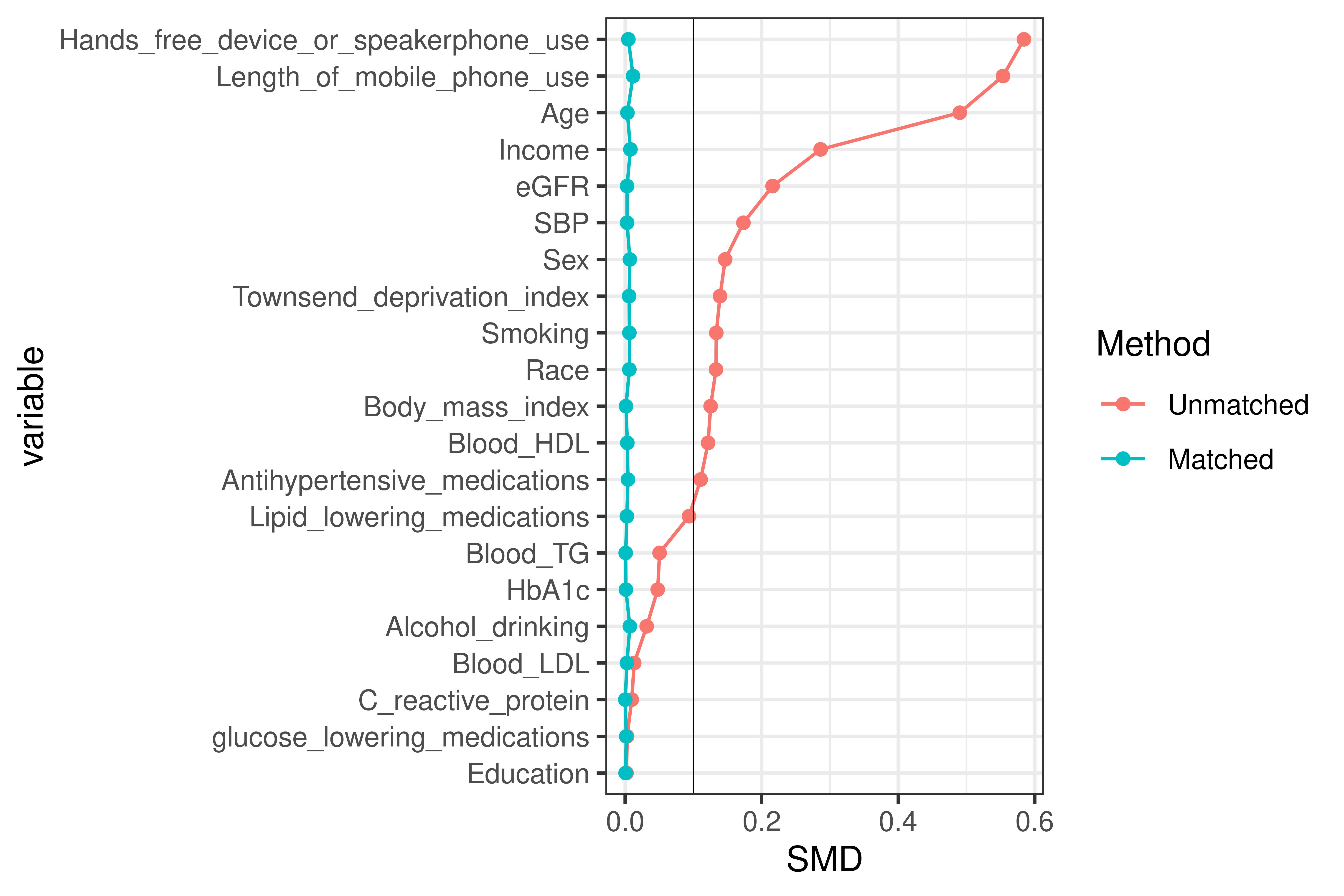
**

**Supplementary Figure 3. Standardized differences of participant characteristics before and after matched according to propensity scores by weekly usage time of mobile phones making or receiving calls (<30mins *vs.* ≥30mins) (UK, 2006-2010)**

**Supplementary Table 1. Disease definitions used in the UK Biobank study (UK, 2006-2010)**

| Disease | **ICD-9** | **ICD-10** | **OPCS-4** |
| --- | --- | --- | --- |
| Chronic kidney disease | 585,5859 | N12.0, N13.1, N13.2, N18.0, N18.3, N18.4, N18.5, N18.8, N18.9 | M01 |

**Abbreviations:** ICD, International Classification of Diseases; OPCS, the Office of Population Censuses and Surveys Classification of Interventions and Procedures.

**Supplementary Table 2. Baseline characteristics of 408,743 participants according to status of mobile phone use (UK, 2006-2010)**

| **Baseline characteristics^*^** | **Mobile phone use** | | | ***P* values** |
| --- | --- | --- | --- | --- |
|  | **No** | **Yes** | |  |
| N | 60141 | 348602 |  | |
| Age, years | 60.2±7.0 | 55.6±8.0 | <0.001 | |
| Male, n (%) | 28483 (47.4) | 160273 (46.0) | <0.001 | |
| White, n (%) | 58299 (96.9) | 331009 (95.0) | <0.001 | |
| Body mass index, kg/m^2^ | 26.8±4.6 | 27.4±4.7 | <0.001 | |
| Systolic blood pressure, mmHg | 141.0±18.8 | 136.7±18.1 | <0.001 | |
| Diastolic blood pressure, mmHg | 82.4±10.0 | 82.1±10.0 | <0.001 | |
| Townsend deprivation index | -1.5±3.0 | -1.4±3.0 | <0.001 | |
| College or University degree, n (%) | 20932 (35.1) | 115492 (33.4) | <0.001 | |
| Smoking status, n (%) |  |  | <0.001 | |
| Never | 36240 (60.5) | 188299 (54.2) |  | |
| Former | 18325 (30.6) | 122626 (35.3) |  | |
| Current | 5382 (9.0) | 36625 (10.5) |  | |
| Alcohol drinking, n (%) |  |  | <0.001 | |
| Never | 6385 (10.6) | 23765 (6.8) |  | |
| <1 time per week | 14947 (24.9) | 75173 (21.6) |  | |
| 1-4 time per week | 26410 (43.9) | 176984 (50.8) |  | |
| Daily or almost daily | 12358 (20.6) | 72487 (20.8) |  | |
| Income, n (%) |  |  | <0.001 | |
| Not to answer/Do not know | 9759 (16.3) | 43377 (12.5) |  | |
| Less than 18,000 | 15431 (25.7) | 60410 (17.4) |  | |
| 18,000 to 30,999 | 15281 (25.5) | 74060 (21.3) |  | |
| 31,000 to 51,999 | 11954 (19.9) | 82559 (23.7) |  | |
| 52,000 to 100,000 | 6522 (10.9) | 68372 (19.7) |  | |
| Greater than 100,000 | 1009 (1.7) | 19114 (5.5) |  | |
| LDL cholesterol, mmol/L | 3.6±0.9 | 3.6±0.9 | <0.001 | |
| HDL cholesterol, mmol/L | 1.5±0.4 | 1.5±0.4 | <0.001 | |
| Triglycerides, mmol/L | 1.7±1.0 | 1.7±1.0 | 0.450 | |
| HbA1c, % | 5.5±0.6 | 5.4±0.6 | <0.001 | |
| C-reactive protein, mg/L | 2.5±4.4 | 2.5±4.1 | 0.001 | |
| eGFR, ml/min/1.73m^2^ | 89.1±11.5 | 92.2±12.0 | <0.001 | |
| Antihypertensive medications use, n (%) | 13971 (23.4) | 63672 (18.4) | <0.001 | |
| Cholesterol lowering medications use, n (%) | 11926 (20) | 53549 (15.5) | <0.001 | |
| Glucose-lowering medications use, n (%) | 2200 (3.7) | 10079 (2.9) | <0.001 | |

^*^The results are presented as Mean ± SD or n (%).

**Abbreviations**: eGFR, estimated glomerular filtration rate; HbA1c, glycosylated hemoglobin, HDL cholesterol, high density lipoprotein cholesterol; LDL cholesterol, low density lipoprotein cholesterol

**Supplementary Table 3. Associations between weekly usage time of mobile phones** **making or receiving calls and new-onset chronic kidney disease with further adjustments for other covariates among 348,602 mobile phone users (UK, 2006-2010)**

| **Weekly usage time of mobile phones making or receiving calls** | **N** | **Events (%)** | **Model 1** | | **Model 2** | |
| --- | --- | --- | --- | --- | --- | --- |
|  |  |  | **HR (95% CI)** | ***P*** value | **HR (95% CI)** | ***P*** value |
| <30 mins | 207820 | 5593(2.7) | Ref |  | Ref |  |
| ≥30 mins | 140782 | 3137(2.2) | 1.12 (1.06,1.19) | <0.001 | 1.12 (1.06,1.19) | <0.001 |

Model 1: adjusted for age, sex, body mass index, ethnicities, Townsend deprivation index, income, education, smoking status, alcohol status, systolic blood pressure, LDL cholesterol, HDL cholesterol, triglycerides, HbA1c, eGFR, C-reactive protein, antihypertensive medications use, cholesterol lowering medications use, glucose-lowering medications use, length of mobile phone use, hands-free device/speakerphone use, physical activity, healthy diet score, self-reported depression, healthy sleep scores and total mental health complaints.

Model2: adjusted for covariates in Model 1 plus genetic risk scores of kidney function.

**Supplementary Table 4. Associations between weekly usage time of mobile phones making or receiving calls and new-onset chronic kidney disease, excluded those who occurred new-onset chronic kidney disease during the first 2 years of follow-up among mobile phone users (UK, 2006-2010)**

| **Weekly usage time of mobile phones making or receiving calls** | **N** | **Events (%)** | **Model 1^*^** | | **Model 2****^†^** | | **Model 3^‡^** | |
| --- | --- | --- | --- | --- | --- | --- | --- | --- |
|  |  |  | **HR (95% CI)** | ***P*** value | **HR (95% CI)** | ***P*** value | **HR (95% CI)** | ***P*** value |
| <30 mins | 207678 | 5451(2.6) | Ref |  | Ref |  | Ref |  |
| ≥30 mins | 140693 | 3048(2.2) | 1.20 (1.15,1.25) | <0.001 | 1.14 (1.08,1.20) | <0.001 | 1.13 (1.07,1.19) | <0.001 |

^*^ Model 1: adjusted for age, sex.

^†^Model 2: adjusted for age, sex, body mass index, ethnicities, Townsend deprivation index, income, education, smoking status, alcohol drinking, systolic blood pressure, LDL cholesterol, HDL cholesterol, triglycerides, HbA1c, eGFR, C-reactive protein, antihypertensive medications use, cholesterol lowering medications use, glucose-lowering medications use.

^‡^Model 3: adjusted for covariates in Model 2 plus mutually adjustments for different behavior of using mobile phone.

**Supplementary Table 5. Relations of diabetes, hypertension and genetic risks of kidney diseases with new-onset chronic kidney disease (UK, 2006-2010)**

|  | | **N** | | | **Events (%)** | | **Model 1^*^** | | | | | | **Model 2^†^** | | | | | |  |
| --- | --- | --- | --- | --- | --- | --- | --- | --- | --- | --- | --- | --- | --- | --- | --- | --- | --- | --- | --- |
|  |  |  |  |  |  |  | **HR (95% CI)** | | | ***P* value** | | | **HR (95% CI)** | | | ***P* value** | | | |
| **Diabetes** | | | |  | | | | |  | | |  | | |  | | |  | |
| No | | | 3365774 | | | 7734(2.3) | | Ref | | |  | | | Ref | | |  | | |
| Yes | | | 20763 | | | 1886(9.1) | | 3.38 (3.21,3.56) | | | <0.001 | | | 2.06 (1.91,2.23) | | | <0.001 | | |
| **Hypertension** | | | |  | | | | |  | | |  | | |  | | |  | |
| No | | | 186409 | | | 2583(1.4) | | Ref | | |  | | | Ref | | |  | | |
| Yes | | | 220681 | | | 8182(3.7) | | 1.82 (1.74,1.91) | | | <0.001 | | | 1.47 (1.37,1.57) | | | <0.001 | | |
| **Genetic risks of kidney diseases** |  | | |  | | | | |  | | |  | | |  | | |  | |
| Low | | | 134765 | | | 2889(2.1) | | Ref | | |  | | | Ref | | |  | | |
| Medium | | | 134765 | | | 3473(2.6) | | 1.20 (1.14,1.26) | | | <0.001 | | | 1.03 (0.97,1.10) | | | 0.284 | | |
| High | | | 134765 | | | 4338(3.2) | | 1.51 (1.44,1.58) | | | <0.001 | | | 1.09 (1.03,1.16) | | | 0.005 | | |

^*^ Model 1: adjusted for age, sex.

^†^Model 2: adjusted for age, sex, body mass index, ethnicities, Townsend deprivation index, income, education, smoking status, alcohol status, systolic blood pressure, LDL cholesterol, HDL cholesterol, triglycerides, HbA1c, eGFR, C-reactive protein, physical activity, self-reported depression, healthy diet scores, healthy sleep scores and total mental health complaints.

**Supplementary Table 6. Associations between mobile phones uses (*vs.* non-users), or weekly usage time of mobile phone making or receiving calls and new-onset chronic kidney disease in the propensity scores matching analysis (UK, 2006-2010)**

| **Mobile phone use**  **behaviors** | **N** | **Events (%)** | **Model 1** | | **Model 2** | |
| --- | --- | --- | --- | --- | --- | --- |
|  |  |  | **HR (95% CI)** | ***P*** value | **HR (95% CI)** | ***P*** value |
| ***Mobile phone users*** | | |  |  |  |  |
| No | 50908 | 1717(3.4) | Ref |  | Ref |  |
| Yes | 50908 | 1840(3.6) | 1.10 (1.03,1.18) | 0.003 | 1.11 (1.04,1.19) | 0.002 |
| ***Weekly usage time of mobile phone making or receiving calls ^*^*** | | |  |  |  |  |
| <30 mins | 91534 | 2092(2.3) | Ref |  | Ref |  |
| ≥30 mins | 91534 | 2194(2.4) | 1.08 (1.02,1.15) | 0.009 | 1.10 (1.03,1.17) | 0.002 |

Model 1: adjusted for age, sex.

Model 2: adjusted for age, sex, body mass index, ethnicities, Townsend deprivation index, income, education, smoking status, alcohol drinking, systolic blood pressure, LDL cholesterol, HDL cholesterol, triglycerides, HbA1c, eGFR, C-reactive protein, antihypertensive medications use, cholesterol lowering medications use, glucose-lowering medications use.

^*^In the analysis, covariates in Model 2 plus mutually adjustments for different characteristics of using mobile phone were adjusted.
